# Death of Prof. Alpheus B. Crosby

**Published:** 1877-10

**Authors:** 


Prof. Alpheus B. Crosby, Professor of Anatomy in Bellevue Hospital Medical College, died from apoplexy, at his country seat, Hanover, N. H., on August 10th. He was born in Gilmington, N. H., in 1832, and was, consequently at the time of his death, forty-five years of age. He received his academic and medical education at Dartmouth College, and shortly after graduation was appointed lecturer, and afterwards Professor of anatomy in that school. In 1871 he was appointed Professor in the Long Island Medical College, and shortly afterwards was elected to the chair of anatomy in the Bellevue Hospital Medical College, which position is made vacant by his death. During the war Dr. Crosby served as surgeon in a New Hampshire regiment, and while stationed in Virginia met his wife, Miss Mildred Smith of Virginia, who with three children survives him. His reputation as a teacher and surgeon, although deservedly great, was scarcely equal to his social popularity, for he was one of the most genial of men. His death will be learned with great regret by all who knew him.—*Maryland Medical Journal.*

